# Placentitis and abortion caused by canine distemper virus in a Linnaeus’s two-toed sloth

**DOI:** 10.1177/10406387261476025

**Published:** 2026-08-29

**Authors:** Jennifer E. Black, Erin Zachar, Maria Bravo-Araya, Davor Ojkic, Jennifer L. Davies

**Affiliations:** Diagnostic Services Unit, Faculty of Veterinary Medicine, University of Calgary, Calgary, AB, Canada; Diagnostic Services Unit, Faculty of Veterinary Medicine, University of Calgary, Calgary, AB, Canada; Prairie Diagnostic Services, Saskatoon, SK, Canada; Animal Health Laboratory, University of Guelph, Guelph, ON, Canada; Diagnostic Services Unit, Faculty of Veterinary Medicine, University of Calgary, Calgary, AB, Canada

**Keywords:** abortion, canine distemper virus, *Choloepus didactylus*, endothelial tropism, placentitis, sloth

## Abstract

Canine distemper virus (CDV; family *Paramyxoviridae*, taxon species *Morbillivirus canis*) causes systemic disease in a broad range of mammalian hosts. CDV commonly targets epithelial, lymphoid, and nervous tissues. Reproductive disease is reported less frequently. Here, we describe CDV-associated placentitis and abortion in a 4-y-old, female Linnaeus’s two-toed sloth (*Choloepus didactylus*) from a private collection. The animal developed respiratory and gastrointestinal illness, aborted a midgestational fetus, and subsequently died. Gross examination revealed necrotizing placentitis. Histologically, the uterus and placenta had vasculitis with thrombosis, endothelial syncytia, and intracytoplasmic and intranuclear viral inclusions within endothelial cells. Additional maternal lesions included bronchointerstitial pneumonia, lymphoid depletion, hepatic necrosis, multisystemic intracytoplasmic and intranuclear viral inclusions, and secondary fungal infection. CDV antigen was abundant within endothelial cells, vessel walls, and perivascular stroma in the uterus and placenta by immunohistochemistry. Real-time PCR detected CDV in the maternal uterus, lung, and placenta. The hemagglutinin gene shared 98.3% nucleotide sequence identity with a CDV previously detected in a raccoon in the United States. Our case documents CDV-associated reproductive failure and endothelial tropism in a sloth, highlighting the potential for severe disease in captive nontraditional hosts.

Canine distemper virus (**CDV**; family *Paramyxoviridae*, taxon species *Morbillivirus canis*) is an enveloped negative-sense RNA morbillivirus. CDV causes canine distemper (**CD**), which is a highly contagious, often fatal, multisystemic disease in domestic dogs and wildlife worldwide.^17,19^ The host range is among the widest of any morbillivirus, with natural infections documented in canids, mustelids, procyonids, ursids, felids, hyenids, viverrids, ailurids, and several noncarnivore species, reflecting the virus’s remarkable ability to cross species barriers.^3,9,17,19^ Infected animals commonly develop immunosuppression along with respiratory, gastrointestinal, cutaneous, and neurologic signs, which reflect the tropism of CDV for lymphoid, epithelial, and nervous tissues.^3,12,17^ CDV spreads primarily through direct contact or aerosol inhalation, and although the virus can cross the placenta, tropism for the reproductive organs, transplacental infections, and fetal loss are rarely reported outcomes in domestic dogs and wildlife.^8,11,15^

Sloths are becoming increasingly popular in North American culture, zoologic collections, and ecotourism. A 2021 review^14^ of infectious agents in two- and three-toed sloths (*Choloepus* spp., *Bradypus* spp.) identified significant knowledge gaps in the prevalence of both clinical and subclinical infectious diseases. The authors emphasized the limited understanding of how infectious diseases affect wild and captive populations and stressed the need for veterinarians to formally report new disease manifestations. Although researchers have documented exposure to a variety of viral, bacterial, fungal, and parasitic agents in sloths, CDV remains the only virus reported to produce clinical disease in either genus.^14^ The first and only reported CDV outbreak in Linnaeus’s two-toed sloths (*Choloepus didactylus*) occurred in 2016 and resulted in fatal multisystemic infection confirmed by histopathology, immunohistochemistry (IHC), virus isolation, and viral sequencing.^16^

We retrieved no reports of CDV-associated reproductive disease or fetal loss in sloths from Google, PubMed, CAB Direct, Web of Science, or Scopus using the search terms “abortion”, “canine distemper virus”, “CDV”, “*Choloepus didactylus*”, “placentitis”, and “sloth”. Here, we expand on the documented spectrum of CDV-associated lesions in sloths and offer evidence of placentitis and abortion in this species.

In July 2023, a male Linnaeus’s two-toed sloth fetus and placenta were submitted to the Diagnostic Services Unit (DSU) at the University of Calgary Faculty of Veterinary Medicine (Calgary, Alberta, Canada) for autopsy. The 4-y-old dam originated from a private zoologic collection and had a 14-d history of hyporexia, upper respiratory tract disease, and diarrhea. Fecal samples submitted to a private diagnostic laboratory tested positive for CDV by PCR.

The fetus was hairless, weighed 166 g, with 13-cm crown–rump length. Based on kidney length (1.2 cm), we estimated the gestational age at 160–180 d (Thielebein J, pers. comm., 2023 Jul 20). Moderate fetal autolysis consistent with in utero death was noted, and gross fetal lesions were absent. The discoid-shaped placenta was edematous, mottled pink to dark-red, with fibrin and necrotic debris coating the chorionic surface, consistent with placentitis (**Fig. 1A**). We collected adrenal glands, brain, esophagus, heart, intestine, kidney, liver, lung, placenta, spleen, testis, thymus, tongue, and trachea for histopathology. We froze samples of placenta, lung, kidney, liver, and spleen at −20°C for future testing. Routine aerobic bacterial culture of placenta, lung, and stomach contents yielded no significant bacterial growth.

**Figure 1.**
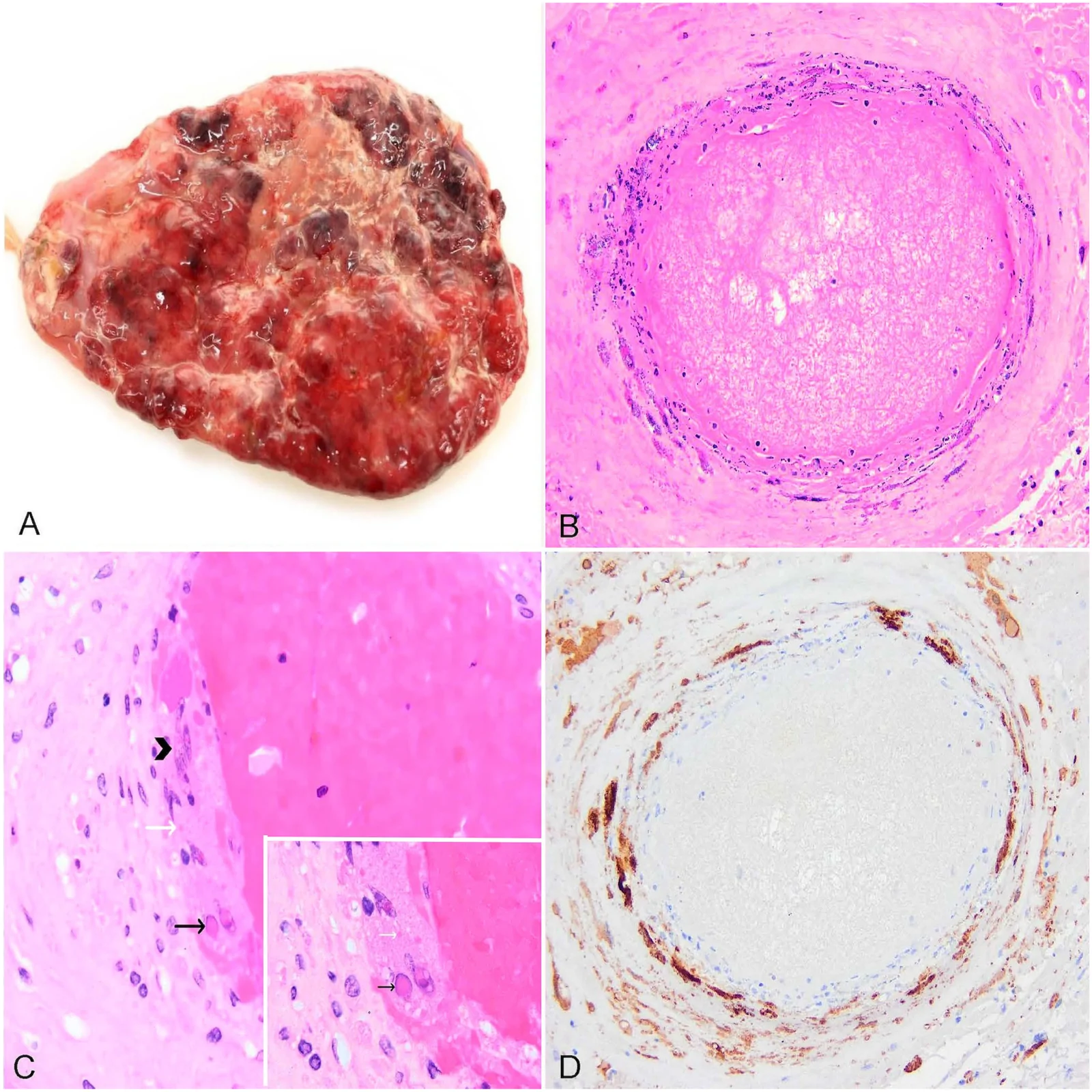
Canine distemper virus (CDV)-associated placentitis in a mid-gestational Linnaeus’s two-toed sloth. **A.** The chorionic surface of the placenta is covered by a layer of tan fibrinonecrotic debris consistent with placentitis. **B.** Necrotizing vasculitis and thrombosis in a placental stromal blood vessel. H&E. **C.** Endothelial cells contain 5–10 μm eosinophilic intracytoplasmic (white arrow) and intranuclear (black arrow) inclusions. Endothelial syncytia are present (arrowhead). Inset: higher magnification. H&E. **D.** Strong immunoreactivity to CDV antigen is localized to endothelial cells within large vessels. Immunohistochemistry.

The dam died 5 d after abortion, and the referring veterinarian (rDVM) performed an autopsy. Gross findings included emaciation along with mild hydropericardium, hydrothorax, and ascites. No other gross lesions were reported. The rDVM submitted formalin-fixed and fresh tissues to the DSU. Formalin-fixed tissues included adrenal glands, brain, colon, diaphragm, esophagus, gallbladder, heart, kidney, jejunum, liver, lung, mesenteric lymph node, pancreas, salivary gland, skeletal muscle, spleen, stomach, thyroid glands, trachea, urinary bladder, and uterus. Fresh samples of lung, liver, kidney, spleen, brain, and uterus were frozen at −20°C.

For histopathology, we fixed tissues in 10% neutral-buffered formalin for 24–48 h, processed them routinely, and stained sections with H&E. We observed no microscopic lesions in fetal tissues. In the placenta, necrotic debris, neutrophils, and fibrin covered the chorionic surface. Necrotizing vasculitis of placental stromal blood vessels included fibrinoid change, neutrophils, dystrophic mineralization, and abundant nuclear debris disrupting the intima and media (**Fig. 1B**). Thrombi frequently occluded affected vessels, and infarcts were present. Endothelial cells contained 5–10 μm eosinophilic intracytoplasmic and intranuclear inclusions, and we observed occasional endothelial syncytia (**Fig. 1C**). We observed identical microscopic vascular lesions in the endometrium and myometrium of the dam.

Additional maternal microscopic lesions included neutrophilic and necrotizing bronchointerstitial pneumonia with rare syncytial cells; severe lymphoid depletion in the spleen and the lymph node; and multifocal, random, hepatic necrosis. Intracytoplasmic and intranuclear viral inclusion bodies were infrequent in bronchial epithelial cells and alveolar histiocytes; abundant in epithelial cells of the trachea, tongue, esophagus, stomach, small intestine, and colon; infrequent in histiocytes of the spleen and the lymph node; and not evident in other tissues. We did not identify lesions in the brain. The glossal, esophageal, and gastric mucosae were covered by innumerable yeasts, pseudohyphae, and hyphae, consistent with secondary fungal infection. *Trichosporon asahii* was considered the likely agent given that it is the predominant cause of opportunistic alimentary mycosis in sloths, particularly in association with CDV infection and other immunosuppressive states.^10^ However, this identification is presumptive given that fungal culture and molecular characterization were not performed because of financial limitations. Notably, *T. asahii* has histologic similarities to *Candida* spp., further complicating identification in the absence of confirmatory culture or molecular methods.

Prairie Diagnostic Services (Saskatoon, SK, Canada) performed IHC using an automated slide stainer (Autostainer Plus; Agilent). Epitope retrieval was performed in a Tris/EDTA pH 9 buffer at 97°C for 20 min. The primary antibody, mouse anti-CDV (clone DV2-12; Custom Monoclonals International), was applied for 30 min, and binding of the primary antibody was detected using an HRP-labeled polymer detection reagent (Agilent). The staining was visualized using 3,3′-diaminobenzidine tetrahydrochloride (DAB; Agilent) as the chromogen, and counterstained with Mayer hematoxylin. Labeling for CDV antigen was detected in the placenta, maternal lung, spleen, liver, small intestine, pancreas, uterus, and adrenal medulla. Labeling intensity was moderate to high, with a granular cytoplasmic pattern in all tissues. In uterus and placenta, strong cytoplasmic labeling was localized to endothelial cells within large vessels (**Fig. 1D**). Following the positive CDV PCR result in fetal lung, IHC was performed, and CDV antigen was not detected. No additional fetal tissues were evaluated by IHC.

The Animal Health Laboratory (Guelph, Ontario, Canada) performed CDV real-time PCR^6^ (rtPCR) and hemagglutinin (*H*) gene sequencing^7^ on placenta, fetal lung, and pooled maternal lung and uterus. All samples tested positive for CDV by rtPCR. Ct values were low in maternal tissues (22.9) and placenta (25.5), consistent with high viral loads, whereas the fetal lung had a comparatively higher Ct value (33.2), indicating a lower viral load. *H* gene sequencing demonstrated 98.3% nucleotide sequence identity to a CDV from a raccoon (*Procyon lotor*) in the United States (GenBank MK577463).^1^

CDV has an exceptionally broad host range, and this promiscuity is relevant to zoological collections, where captive conditions and the presence of multiple mammalian species increase the risk of CDV transmission.^9^ CD has been reported only once in sloths, in a fatal outbreak affecting captive, adult, Linnaeus’s two-toed sloths caused by the American-4 strain.^16^ Like that report, microscopic lesions in our case included bronchointerstitial pneumonia, lymphoid depletion, intracytoplasmic and intranuclear viral inclusions within epithelial cells and histiocytes, syncytial cell formation, and widespread viral antigen distribution. Multifocal random hepatic necrosis was prominent in our case, suggestive of a potential hepatic tropism previously reported in this species. The basis for hepatic involvement in sloths remains unclear but may reflect either host-specific susceptibility or CDV strain variability.

Reproductive failure associated with CDV infection has not been reported in sloths, and our case expands the recognized spectrum of CDV-associated disease in this species to include direct involvement of the maternal–fetal interface. Across susceptible hosts, reproductive manifestations of CDV infection are considered uncommon. In domestic dogs, transplacental transmission has been documented, resulting in abortion, weak neonates, or neonatal mortality associated with lesions such as thymic atrophy, encephalitis, and interstitial pneumonia.^8,11^ Vertical transmission has also been reported in a wild ferret, raising the possibility that transplacental infection contributes to viral maintenance within wildlife populations.^15^ Comparable transplacental infection occurs with other morbilliviruses, including the measles virus in humans and phocine distemper virus in marine mammals.^4,5^

In our case, although CDV antigen was definitively identified in the uterine and placental lesions, evidence supporting vertical transmission was inconclusive. The detection of CDV nucleic acid in fetal lung by PCR may support fetal infection. However, given the absence of microscopic lesions, the high Ct value (33.2), and the negative IHC labeling in the fetal lung, contamination of the fetal tissue at autopsy remains a plausible alternative explanation. Nonetheless, considering the increasing reliance on captive breeding programs for threatened species, reproductive failure associated with CDV infection—especially in a species not previously recognized to experience this manifestation—is concerning, particularly as the host range of this virus broadens.

An unusual feature of our case was marked tropism of CDV for reproductive endothelial cells. The H protein mediates viral attachment and determines tissue tropism. Classically, CDV is lymphotropic, epitheliotropic, and neurotropic, mediated by SLAM/CD150 in immune cells and nectin-4 in epithelial cells, with neurotropism also involving an as-yet uncharacterized receptor.^12,20^ Endothelial infection occurs mainly in advanced systemic disease and is generally attributed to high-level viremia rather than primary endothelial tropism; accordingly, vascular lesions are not typical of CDV infection.^2,13,20^ The severe vascular lesions in our case invite comparison with endotheliotropic henipaviruses, which target endothelial cells via ephrin B2, resulting in widespread vascular injury and, in some models, placental infection and fetal loss.^18^ CDV has not been shown to use ephrin receptors, and mechanisms of endothelial entry remain unclear.^12,20^ The endothelial tropism in our case may reflect host–virus interactions, strain-specific features, overwhelming viremia, or the possibility that CDV exploits alternative receptors under certain conditions.

We had limited clinical history for our case and lacked information on biosecurity measures, surveillance practices, vaccination status, and the presence of other susceptible species in the collection, limiting our ability to determine the source of infection. We also did not perform environmental or cohort testing to further investigate potential sources of exposure.

Although we could not definitively identify the source of CDV introduction in our case, spillover from free-ranging wildlife, especially raccoons, remains a plausible explanation. In North America, sylvatic and urban raccoons are widely recognized as important reservoir hosts and play a central role in maintaining viral circulation, genetic diversity, and cross-species transmission, including spillover into captive and nondomestic species.^1,3,17,19^ In eastern Canada, one study demonstrated that CDV lineages co-circulate among free-ranging carnivores, with raccoons functioning as key maintenance and amplification hosts.^7^ In contrast, comparable strain level data from western Canada, including Alberta, is limited, leaving gaps in our understanding of regional CDV epidemiology and transmission dynamics.

Overall, our case underscores the continued risk posed by endemic CDV circulation to zoologic collections and nontraditional species, emphasizing the need for rigorous biosecurity and expanded sequence-based surveillance to better define regional CDV epidemiology for risk analysis and evidence-based management strategies.
